# Clinical, socio-demographic and radiological predictors of short-term outcome in rotator cuff disease

**DOI:** 10.1186/1471-2474-11-239

**Published:** 2010-10-15

**Authors:** Ole M Ekeberg, Erik Bautz-Holter, Niels G Juel, Kaia Engebretsen, Synnøve Kvalheim, Jens I Brox

**Affiliations:** 1 Department of Physical Medicine and Rehabilitation, Oslo University Hospital Ullevål, Oslo, Norway; 2 Medical Faculty, University of Oslo, Oslo, Norway; 3 Department of Orthopaedic Surgery, Section of Physical Medicine and Rehabilitation, Oslo University Hospital, Rikshospitalet and Medical Faculty University of Oslo, Oslo, Norway

## Abstract

**Background:**

Shoulder pain is common with rotator cuff disease as the most frequently used clinical diagnosis. There is a wide range of treatment options for this condition, but limited evidence to guide patients and clinicians in the choice of treatment strategy. The purpose of this study was to investigate possible prognostic factors of short-term outcome after corticosteroid injection for rotator cuff disease.

**Methods:**

We performed analyses of data from 104 patients who had participated in a randomized controlled study. Socio-demographic, clinical and radiographic baseline factors were assessed for association with outcome at six-weeks follow-up evaluated by Shoulder Pain and Disability Index (SPADI) and patient perceived outcome. Factors with significant univariate association were entered into multivariate linear and logistic regression analyses.

**Results:**

In the multivariate analyses; a high SPADI score indicating pain and disability at follow-up was associated with decreasing age, male gender, high baseline pain and disability, being on sick-leave, and using regular pain medication. A successful patient perceived outcome was associated with not being on sick-leave, high active abduction, local corticosteroid injection and previous cortisone injections. Structural findings of rotator cuff tendon pathology on MRI and bursal exudation or thickening on ultrasonography did not contribute to the predictive model.

**Conclusions:**

Baseline characteristics were associated with outcome after corticosteroid injection in rotator cuff disease. Sick-leave was the best predictor of poor short-term outcome. Trial registration: Clinical trials NCT00640575

## Background

Shoulder pain is common with a one-year prevalence reported up to 47% in a general population [1,2]. A large proportion of patients with shoulder pain have an unfavourable outcome with long-term disability [3]. Recent high quality studies of prognostic factors may prove valuable predicting the likely course of outcome in the individual patient [4,5]. Long duration of symptoms, more intense pain, other musculoskeletal symptoms, and psychosocial factors have been identified as predictors of poor outcome [6]. Still little evidence exists to guide patients and clinicians in treatment selection.

Subacromial impingement syndrome or rotator cuff disease is the most frequently used clinical shoulder diagnosis [7,8]. Patients present pain predominantly in the lateral deltoid area, which increases on elevation. Passive glenohumeral mobility is within the normal range and a Neer or Hawkins-Kennedy impingement sign will elicit pain [9]. The clinical diagnosis may not reveal the underlying pathoanatomical changes, which may span from subacromial bursitis, tendinopathy with or without calcifications to partial and full-thickness rotator cuff ruptures. Advanced radiological modalities like diagnostic ultrasonography and magnetic resonance imaging (MRI) are reliable in detecting structural abnormalities in the rotator cuff [10]. The exact mechanism of pain is poorly understood [11] and structural changes are found increasing with age also in asymptomatic subjects [12-15]. It is largely unknown to what extent structural findings identified by routine imaging affect outcome of non-operative treatment of shoulder pain. The costs of implementing advanced radiology for diagnostics are considerable. Currently, the evidence for choosing a treatment strategy based on pathological findings is scant.

The purpose of the present study was to assess to what extent socio-demographic factors, occupational factors and clinical and radiological findings predicted outcome in patients with rotator cuff disease receiving corticosteroid injection treatment. We hypothesized that baseline socio-demographic factors and patient reported pain and disability were moderately associated with outcome at six weeks, and that structural findings identified by routine MRI and ultrasonography would contribute to the prognostic model.

## Methods

We performed an analysis of data from a randomized controlled trial comparing local and systemic corticosteroid injection in rotator cuff disease [16].

### Study population

One hundred and six patients with a clinical diagnosis of rotator cuff disease had been randomized six weeks previously to systemic or ultrasonographically guided corticosteroid injection. Two patients were not available for follow-up at six weeks and were excluded from analyses in the present study. We reported previously that no difference was found between systemic and local corticosteroid injection at six-weeks follow-up. A detailed description of patients, treatments and concomitant treatment is reported in that paper [16]. The committee for medical research ethics in Norway approved the project.

### Outcome measures and predictors

Primary outcome was the Shoulder Pain and Disability Index (SPADI) [17] measured at six-weeks follow-up. Secondary outcome was a patient reported global assessment score measured at six-weeks follow-up [18]. SPADI is a validated self-report shoulder questionnaire consisting of five pain and eight disability items, giving a total sum score ranging from 0 (best) to 100 (worst). The global assessment score consisted of four levels of patient perceived judgement of overall shoulder complaint, ranging from poor to excellent. Reporting a good or excellent overall shoulder condition was regarded as a successful outcome.

All patients filled in a standardized questionnaire including the potential prognostic factors and outcome measures and underwent a clinical examination. The same physician examined all patients at baseline and follow-up. Patients were asked to rate their pain in activity from 0 (no pain) to 9 (severe pain), the highest level of professional education (primary school/secondary school/university), work status (not working/working or partly working), sick-leave (no/yes or part time), pain medication (daily or weekly/less often than weekly/seldom/never), duration of shoulder complaint (3-6 months, 6-12 months, 12-24 months, more than 24 months) concomitant neck pain (yes/no), previous episodes of shoulder pain (yes/no), dominant arm affected (yes/no), bilateral shoulder pain (yes/no), beliefs of precipitating cause of shoulder pain (minor trauma/overuse/stress/unknown), previous treatment (physiotherapy/corticosteroid injections). Emotional distress was measured using the 25-item Hopkins symptom check list (HSCL-25) [19]. Patients were considered distressed if the average item score exceeded 1.74, which has been found to be a good predictor of current help-seeking in a Norwegian epidemiologic study [20]. Self-efficacy for pain was evaluated using four seven-point ordinal scales [21]. Patient's expectancies of treatment effect were evaluated using three items considering the expected outcome of the overall shoulder problem; the shoulder pain and; the ability to use and move the shoulder measured on a seven-point ordinal scale with verbal anchors ranging from much worse to much better [22].

We interviewed employed patients about work tasks (categorized as manual or not manual work). Patients evaluated statements about the amount of time they were lifting heavy objects (10 kg), working with hands above shoulder level, or conducting repetitive tasks on six-point ordinal scales ranging from "all the time" to "never". Patients reported perceived physical work load, occupational stress, job satisfaction, and comfort with co-workers and supervisors at work on six-point ordinal scales ranging from "extreme" to "not at all".

Descriptions from routine MRI were recorded for evaluating the influence of MRI verified rotator cuff ruptures and presence of calcifications in the rotator cuff on outcome. MRIs taken before referral to the outpatient clinic were accepted if images were obtained within the last three months. Data were extracted from the radiologist's evaluation. Rotator cuff tendons were categorized as normal, with tendinopathy, partial-thickness and full-thickness ruptures. Calcifications were registered as present or non-present. Eight patients were unwilling to be examined by MRI and had diagnostic ultrasound examination. The presence of bursal exudates and thickening of the bursal walls were recorded by diagnostic ultrasound in all patients before injection treatment by visual estimation (present/not present) by the consultant physician providing the injections.

Active ranges of abduction and flexion were measured with a handheld digital inclinometer according to a standardized protocol [23]. The effect of 5 ml of 1% lignocaine hydrochloride injected into the subacromial bursa was measured by 100 mm visual analogue scales of pain; at elevation; isometric abduction; Hawkins-Kennedy and Neer impingement sign before and five minutes after injection and by overall patient rated improvement in categories no, moderate or much improvement. A reduction of more than 50% of the sum score of the four visual analogue scales was evaluated as a positive impingement test. The complete set of visual analogue scales before and after injections were missing in eight patients and for these we used the perceived overall rating categorized as much improvement as a positive impingement test.

### Statistical analysis

We did a secondary analysis of a randomized clinical trial; hence no formal sample size calculation was performed before starting the study. There is no straightforward method of estimating sample size in multivariable prognostic research. We required at least 10 outcomes for each predictor studied [24].

Univariate and multivariate linear and logistic regression analyses were used to investigate the associations between potential prognostic variables and short-term outcome after injection treatment. Factors showing a univariate association with the outcome at issue (p < 0.2) were extracted as candidate variables to be used in the multivariate analysis.

In the multivariate linear model, all potential predictors were entered into the multivariate model. Adjustments were made for gender and treatment group. Variables not statistically associated with the dependent variable on p < 0.05-level were eliminated using a manual backward stepwise technique except for gender and treatment group that were kept regardless of significance level. We used an additional sequential block model to investigate the predictive power of structural pathology measured by routine MRI and ultrasonography on outcome of shoulder pain and disability.

We used a stepwise forward technique in the multivariate logistic regression model because not more than one variable per 10 patients in each group were allowed simultaneously in the model.

Predictors that are highly correlated contribute little independent information. We included the clinically most important predictor if r > 0.7 between two predictors. Continuous variables were checked for linearity using curve estimation plots. Models were checked for normality, homoscedasticity and colinearity by inspecting residuals and calculating variance inflation factor (VIF) [25]. We used Chi-square statistics to evaluate associations between gender, sick-leave, occupational factors and self perceived outcome in the subpopulation of employed patients. All p-values are two sided with significance level of 5%. Analyses were carried out using SPSS for Mac 16.0.1.

## Results

Table 1 presents the baseline characteristics of the study population. Patients reporting good or excellent condition on the four-point global assessment of shoulder complaint at follow-up did not differ in age, gender, duration or severity of shoulder disease compared to patients reporting moderate or poor shoulder condition. We found one case that represented a possible outlier in both the linear and logistic regression model with a standardized residual above three. Excluding the case did not influence results and the case was kept in the final analysis. Influence and leverage statistics were within normal range and there were no signs of colinearity. Models assumptions were judged as met.

**Table 1:** Baseline characteristics

	Overall (n = 104)	Good or excellent (n = 42)	Poor or moderate (n = 62)
Age, mean (SD)	52 (12)	53 (11)	49 (11)

Women	63 (61)	25 (60)	38 (61)

Duration of complaints			

<6 months	30 (30)	13 (31)	17 (27)

6 to 12 months	32 (24)	11 (26)	21 (34)

12 to 24 months	17 (22)	7 (17)	10 (16)

more than 24 months	25 (24)	11 (26)	14 (23)

Baseline SPADI score, mean (SD)	52 (18)	49 (19)	54 (17)

Baseline pain in activity, mean (SD)	6.2 (1.5)	6.0 (1.4)	6.4 (1.6)

Baseline characteristics of the overall study population and grouped according to the four-point global assessment of shoulder complaint at six-weeks follow-up. Values are numbers (percentages) unless stated otherwise.

### Associations with outcome at six-weeks follow-up

At baseline, mean SPADI score was 52 points, 51 points in the systemic group and 53 points in the local group. At six-weeks follow-up this was reduced to 31 points, 32 points in the systemic group and 29 points in the local group. There were no gender differences in SPADI score at baseline, but at six-weeks follow-up there was a lower SPADI score in females (28 points) than males (35 points). The overall improvement observed at follow-up in both groups, exceeds the minimal clinical important change in SPADI [26]. Independent univariate factors associated with follow-up SPADI score and missing values for each variable are reported in table 2. Two patients had incomplete responses to one or more prognostic variables, leaving 102 patients (98%) for the final multivariate linear regression analysis. Results from the multivariate linear analysis are shown in table 3. Age, gender, baseline SPADI score, regular pain medication and previous episodes of shoulder pain were significantly associated with follow up SPADI score. The model explained 40 percent of the variance in the follow-up SPADI score. Men scored on average 14 points (95% CI 6.4 to 22.6) higher than women at follow-up and patients using regular pain medication at baseline scored on average nine points (95% CI 1.0 to 17.7) more on follow-up SPADI score than patients not using regular pain medication at six-weeks follow-up. There was no interaction between gender and baseline SPADI score. Increasing age resulted in a lower SPADI follow-up score with a factor of approximately 0.4 point (95% CI -0.8 to -0.1) per year. The predictors; previous episodes of shoulder pain, a restricted active abduction at baseline, and a high baseline SPADI score, were associated with outcome at follow up. Baseline emotional distress, self-efficacy for pain, and outcome expectations were not significantly associated with outcome. The radiological variables; structural findings of rotator cuff tendon pathology (tendinopathy, partial or full thickness ruptures and calcifications), bursal exudation and thickening on sonography, did not contribute to the predictive model (R^2 ^change = 0.03, p = 0.68). Factors with a univariate association with the patient perceived shoulder condition at six-weeks follow-up are reported in table 2. Not being on sick-leave; high baseline active abduction, local corticosteroid injection and previous corticosteroid injection were significantly associated with a successful outcome in the multivariate logistic regression analysis.

**Table 2:** Univariate associations

			SPADI Univariate linear regression			Global assessment score Univariate logistic regression		
Variable	value*	missing, n	beta	95% CI	p-value	OR	95% CI	p-value

Age, mean (SD)	51 (11)	0	-0.3	-0.8 to 0.1	0.10	1.03	0.99 to 1.07	0.08

Gender, female	63	0	7.0	-2.4 to 16.3	0.14	1.08	0.48 to 2.40	0.86

Education More than 12 years	42	1	1.4	-8.1 to 10.9	0.78	0.91	0.41 to 2.05	0.83

On sick leave	30	0	14.0	4.3 to 23.8	0.01	0.34	0.13 to 0.89	0.03

Duration of complaints, 3-6 months vs.	30							
6-12 months	32		4.6	-7.4 to 16.7	0.45	0.69	0.25 to 1.91	0.47
12-24 months	17		0.5	-13.9 to 14.8	0.95	0.92	0,27 to 3,06	0.88
more than 24 months	25	0	4.0	-8.9 to 16.8	0.54	1.03	0,35 to 3,00	0.96

Precipitating cause, unknown vs	45							
minor trauma	14		-11.2	-25.1 to 2.7	0.11	6.15	1.63 to 23.19	0.07
Overuse	43	2	-3.0	-13.4 to 7.3	0.56	1.77	0.73 to 4.29	0.21

Previous episodes of shoulder pain	68	0	6.7	-3.2 to 16.5	0.18	1.06	0.46 to 2.47	0.88

Concomitant neck pain	64	0	0.4	-9.1 to 9.9	0.93	1.03	0.46 to 2.29	0.95

Dominant side affected	67	0	9.1	-0.33 to 18.59	0.058	0.50	0.22 to 1.12	0.09

Baseline SPADI score, mean (SD)	52 (18)	0	0.57	0.34 to 0.81	0.001	0.98	0.96 to 1.01	0.16

Baseline pain in activity, mean (SD)	6.2 (1.5)	0	4.7	1.8 to 7.6	0.002	0.82	0.63 to 1.07	0.14

Regular pain medication	33	0	13.3	3.8 to 22.9	0.007	0.53	0.22 to 1.27	0.16

Previous corticosteroid	42	0	-5.0	-14.4 to 4.3	0.29	2.30	1.03 to 5.20	0.04

Bilateral shoulder complaint	17	0	1.3	-11.2 to 13.7	0.84	1.84	0.65 to 5.24	0.25

Active abduction, mean degrees (SD)	118 (33)	0	-0.21	-0.34 to 0.07	0.03	1.02	1.00 to 1.03	0.02

Impingement test, positive	52	0	-15.4	-24.3 to -6.5	0.001	1.99	0.89 to 4.40	0.09

Rotator cuff, normal versus	30							
tendinopathy	44		-7.0	-18.1 to 4.1		0.83	0.32 to 2.20	0.71
partial rupture	22		-7.6	-20.6 to 5.5	0.25	1.25	0.41 to 3,80	0.69
full-thickness rupture	10	0	-17.3	-34.3 to 0.3	0.05	1.50	0.36 to 6.32	0.58

Calcification	25	0	5.0	-5.7 to 15.8	0.36	0.98	0.39 to 2.45	0.96

Bursal exudation	54	2	-2.0	-11.4 to 7.4	0.68	0.82	0.37 to 1.80	0.62

Bursal thickening	64	2	-2.3	-12.0 to 7.4	0.64	0.94	0.42 to 2.12	0.88

Treatment group, local corticosteroid	52	0	-3.0	-12.2 to 6.2	0.52	2.49	1.11 to 5.60	0.03

Distress	28	8	7.4	-3.0 to 17.8	0.16	0.79	0.32 to 1.98	0.62

Positive outcome expectations	67	6	2.3	-8.0 to 12.6	0.66	0.94	0,83 to 1.07	0.35

Self-efficiency for pain, mean (SD)	15.2 (4.6)	5	0.9	-0.2 to 1.9	0.1	0.92	0.84 to 1.01	0.08

* numbers of subjects unless stated otherwise.

Univariate associations between socio-demographic, clinical and radiological variables and outcome measured by SPADI (linear regression) and global assessment score (excellent or good vs. moderate or poor) (logistic regression) measured six weeks after corticosteroid injection therapy.

**Table 3:** Multivariate linear regression model.

	**R**^**2**^	B	95% CI	p-value
**Step 1**	0.401			<0.001

Age, years		-0.4	-0.8 to -0.1	0.024

Gender male		14.2	6.4 to 22.6	0.001

Baseline SPADI score		0.4	0.2 to 0.7	0.001

Treatment group, local injection		-4.8	-12.7 to 2.5	0.186

Regular pain medication		9.1	1.0 to 17.7	0.029

Total abduction at baseline		-0.1	-0.3 to -0.0	0.031

Previous episodes of shoulder pain		8.8	0.3 to 17.0	0.042

On sick leave		10.3	1.2 to 19.5	0.027

**Step 2**	0.427			0.68

Rotator cuff, normal vs.				

Tendinopathy		3.9	-7.2 to 14.9	0.49

Partial thickness tears		-2.9	-15.3 to 9.5	0.29

Full-thickness tears		-8.8	-25.1 to 7.5	0.53

Calcification		-3.2	-13.4 to 7.0	0.53

Bursal effusion		1.5	-7.8 to 10.7	0.76

Bursal thickening		-3.9	-14.0 to 6.1	0.44

Total SPADI score at six weeks after corticosteroid injection treatment as dependent variable and associated baseline predictors after stepwise backward selection. Model 2 illustrates the additional predictive ability of including variables from routine radiology examination.

### Occupational factors

Among employed patients (n = 86), significantly more men had manual labour (χ2 = 13.7, p < 0,001). There was no association between gender and the other occupational variables included. Patients on sick-leave experienced their work as more physically demanding (χ2 = 8.6, p = 0.003), reported more frequently working above shoulder level (χ2 = 13.3, p = 0.001) and lifting heavy objects (χ2 = 7.5, p = 0.006). Frequent work with arms above shoulder level was associated with a poor or moderate outcome (χ2 = 5.5, p = 0.02). Other measured occupational factors were not significantly associated with outcome. Level of education did not influence outcome, but was associated with sick-leave (χ2 = 8.5, p = 0.004) and overhead work (χ2 = 8.24, p = 0.04).

## Discussion

The present study confirmed that socio-demographic factors and patient reported pain and disability are strong predictors of outcome. Structural findings in rotator cuff and subacromial bursa did not contribute to predict outcome. There might be several reasons for these findings.

Sick-leave and low active abduction were identified as negative predictors by evaluating outcome both by SPADI and the patient perceived global assessment, and may be regarded as the most robust findings in the present study. The difference in construct measured by the two outcome measures and the statistic model applied are likely to influence the results. In addition to sick-leave; regular pain medication; previous episodes of shoulder pain and gender were the strongest predictors for perceived pain and disability six weeks after treatment. The sick-listing process is complex; both work related (physical and psychosocial) and individual factors like comorbidities and coping ability are associated with sick-leave and health care use [27]. A longitudinal study reported that female patients more often took sick-leave for neck and shoulder complaints [28]. Sick-leave and use of pain medication have previously been recognized as risk factors of poor short-term and long-term outcome after supervised exercises and surgery in patients with subacromial pain [18,29]. Pharmacological, physical and mental mechanisms may contribute. Patients using pain medication (NSAIDs and painkillers) may be less likely to have an additional anti-inflammatory effect of corticosteroids and patients having physically demanding work are more likely to be on sick-leave. Contrary, sick-leave and pain medication may be regarded as passive coping mechanisms. Sick-leave and pain medication are socio-medico-legally-cultural accepted, and may be introduced or at least reinforced by the physician. It is a paradox that sick-leave and pain medication when controlled for in the multivariate analyses, contribute to poorer short-term prognosis. We may hypothesise that a more restrictive prescription of sick leave and pain medication may contribute to improved prognosis.

The observed association between SPADI and gender in the present study was unexpected. There is a higher prevalence of musculoskeletal disorders among women based on self-reports [30,31] and women seem to seek health care providers and use medication more frequently than men [32]. There is limited evidence for gender differences in outcome after treatment for shoulder disease [33]. Thomas et al. reported that male gender was associated with poor perceived long term outcome after nonoperative treatment for shoulder pain in general practice [34]. In the present study contradictory results were obtained using the logistic model with a perceived global outcome. A possible explanation is that the item selection in the SPADI score fits male patients better than females [35]. Because of the small sample size, including a high proportion of women and that other factors associated with outcome and gender were not assessed in the present study, we cannot exclude that the association with gender and SPADI score is spurious. By example, more men had manual labour. The method of data collection and the relatively small sample size did not allow for a thorough analysis of work related factors. The observed differences between genders in response to treatment support stratification on gender in clinical trials of shoulder disease.

In the present study, structural changes in rotator cuff and subacromial bursa on routine radiological examination did not predict short-term outcome after corticosteroid injection therapy. There is an ongoing debate regarding aetiology and pain mechanisms in rotator cuff disease. Both extrinsic subacromial impingement and intrinsic tendon degeneration from age, genetic disposition and tensile overload within the rotator cuff tendons are regarded as mechanisms resulting in rotator cuff disease. The subacromial bursa has been identified as a key structure mediating pain [36-38], though pain may also arise from within the tendon itself [39,40]. The inconsistency in the literature and in clinical practice on efficacy of corticosteroid injections in rotator cuff disease may relate to subgroups of patients with different histological findings [41]. Fibrosis of the subacromial bursa, size of rotator cuff tear and degenerative findings like spurs under the acromion suggest worse long term outcome after physiotherapy, injections and surgery [42-46]. Still, the evidence for choosing a treatment strategy based on pathological finding is scant. Surgical treatment of rotator cuff tears is considered a valid therapeutic approach because size of the rotator cuff tears, degree of atrophy and fatty degeneration influence glenohumeral kinematics, progress over time and are negative predictors of outcome after rotator cuff surgery [47-49]. Further, surgical management of small and medium rotator cuff tears (< 3 cm) have been reported superior to physiotherapy in one randomized study [50]. Structural changes are, however, found in asymptomatic shoulders and tendinopathy and rotator cuff tears are associated with increasing age [12-15]. Tear size exceeding 3 cm in the medial to lateral plane, signs of atrophy and fatty degeneration is reported associated with symptomatic rotator cuff tears, but to reliably differentiate between symptomatic and asymptomatic structural changes based on radiology is still an unsolved challenge [51]. As evident in other musculoskeletal disorders, uncritical use of radiological modalities may result in labelling patients with an anatomic diagnosis that might not be the actual cause of symptoms and potentially unjustified treatments [52,53]. A structural diagnosis should be supplemental to careful clinical testing. Clinical examination is sensitive for detection of rotator cuff tears [54].

There are several limitations of the present study. First, the sample size is small which tend to over fit the predictive model to the data and spuriously overestimate associations between factors and outcome [55]. Secondly, the short follow up period reduces the application of study findings. Yet, corticosteroid injection is often adjunct to other treatments like supervised exercises and short-term pain relief is often the treatment goal. We used radiologists' reports of routine MRI for assessing integrity of the rotator cuff. Differences in MRI protocols, radiologists' assessment criteria and inter- and intratester reliability have not been assessed in the present study. Likewise, we have not evaluated the reliability and validity of the ultrasound assessment used in the present study. These factors may have affected the structural diagnosis reported and thus biased our results. The radiologists did neither assess the size of the rotator cuff tears, nor the degree of atrophy, fatty degeneration of the rotator cuff muscles, and rotator interval lesions. Our results should be interpreted with caution. However, the strategy applied reflects clinical practice, as the clinician will have to accept differences in MRI protocols and reports from generic radiologists. Despite the limitation of the findings in the present study, we question the usefulness of routine imaging in the initial examination of patients with shoulder pain. Randomized trials in patients with low back pain [56] and low back pain and sciatica [57] have found that early use of imaging do not affect prognosis. A future study in patients with shoulder pain should evaluate the efficacy of early MRI imaging using a randomized design. An optimal predefined protocol should be applied for assessment of specific findings and examine whether full-thickness and partial-thickness ruptures or other diagnoses predict short-and long-term clinical outcome.

## Conclusion

Baseline characteristics are predictors of outcome after corticosteroid injection treatment in rotator cuff disease. Sick-leave was the best predictor of poor short-term prognosis. Findings from routine radiological examination did not predict outcome.

## Competing interests

The authors declare that they have no competing interests.

## Authors' contributions

OME, KE and JIB designed the study. OME recruited the patients in contributions with NGJ, SK and EBH. OME, KE and JIB performed the statistical analysis. OME drafted the manuscript together with KE, JIB, SK, NGJ and EBH. All authors read and approved the final manuscript.

## Pre-publication history

The pre-publication history for this paper can be accessed here:

http://www.biomedcentral.com/1471-2474/11/239/prepub
